# On the Exhibition of Food in Typhoid Fever

**Published:** 1862-09

**Authors:** 


### CHICAGO
### MEDICAL JOURNAL.

VOL. VI.] SEPTEMBER, 1862. [No. 9.

### SELECTED.

### ON THE EXHIBITION OF FOOD IN TYPHOID FEVER.

The following remarks made by Mr. Herard, in a clinical lecture at the Hotel Dieu, at Paris, are of particular interest at the present time, especially to the medical officers of our army hospitals:—

"The treatment of typhoid fever is of course different according to the theory adopted on the nature of the disease. The practitioner who views in typhoid fever follicular inflammation of the intestinal tube, an ulcerous affection of Peyer's glands, and consequent absorption of poisonous fluids calculated to induce a septic condition, consistently prescribes antiphlogistic remedies in the incipient stage, and tonics in the more advanced period of the disease. Likewise, those who conceive that the decomposition of the local secretions is the primary cause of the infection of the system act consonantly with their theory in systematically exhibiting emeto-cathartics and laxatives. In these opinions, however, M. Herard does not participate; while taking into serious account the intestinal eruption, which, like that of smallpox, induces a certain amount of circumambient inflammation, he cannot admit this to be the proximate cause of typhoid fever. No concordance can be traced between this anatomical change and the general condition of the patient, the gravity of which must be acknowledged to be entirely independent of the local injury. Hence the latter cannot be taken for a guide in the choice of the medication most appropriate to a fever in which the collapse of vital power and the obvious tendency of hemorrhage and mortification point most distinctly to a primary alteration in the composition of the blood. M. Herard does not deny that an emeto-cathartic may be proper to remedy the foul state of the primæ viæ, so common in the early stage of typhoid, but he can neither concede to aperients, to venesection, nor to local bloodletting, the privilege of being the exclusively appropriate modes of treatment of the disease.

"In typhoid fever M. Herard proceeds as follows :

"In moderate, and *à fortiori* in mild cases, he refrains from any active interference calculated to debilitate the patient, and to cause the disease to assume the dangerous form which justly occasions so much dread. M. Herard prescribes an emeto-cathartic, one or two doses of saline aperients, a few baths if the skin be very hot and dry, and wine and water. Baths restore the functions of the skin, and usually induce sleep. In the adynamic variety he resorts to tonics, stimulates the system with Malaga or Bordeaux wine, either in drinks or in enemas, prescribes from half a drachm to a drachm of powdered cinchona bark, in coffee without milk, and also recommends various stimulants, such as musk, camphor, acetate and carbonate of ammonia. He causes, at the same time, the eschars to be covered with powdered Peruvian bark, and requires from the nurses the most strict attention to cleanliness. In the ataxic form, the most fatal of all, bloodletting, leeches, blisters, are unavailing ; all remedies seem powerless. In order, however, not to appear inactive in cases of such dire gravity, he prescribes stimulants, dry cupping of the extremities, blisters to the nape of the neck, and cold effusions cautiously administered. In the thoracic form, which this year has been the most prevalent, blistering and cupping, with scarification, are the remedies which M. Herard has chiefly resorted.

"The above is a brief summary of the treatment appropriate to typhoid ; but in the management of the disease the all-important, the capital question is that of food.

"Despite the wise precepts of Hippocrates, said M. Herard, despite the recent researches which have only confirmed their value, we are still all more or less influenced by the now exploded doctrine of irritation. The terms fever and food still appear to imply a contradiction, although it is but too certain that in typhoid prolonged abstinence leads to the most desastrous results.

"Some ten years ago, M. Herard was in attendance on a lady suffering from a moderately violent attack of the malady under consideration. Cerebral symptoms having set in, a consultation took place, and an eminent professor of the school of medicine recommended absolute abstinence from food, and the daily exhibition of one or two glasses of seidlitz water. The latter part of the prescription M. Herard took upon himself in some degree to modify, but the abstinence was strictly enforced. After two or three weeks' treatment, the pulse rose from 110 to 120, nocturnal agitation set in, with wandering, delirium, vomiting, and diarrhœa. On the following days the frequency of the pulse increased to 145, vomiting became incessant, the diarrhœa incoercible, the delirium constant; the tongue was red, and thrush appeared over the entire mucous lining of the mouth. Another consultation was deemed expedient; the three gentlemen whose opinion was requested viewed the case in a different light. One pronounced the patient to be suffering from softening of the stomach; the others, struck by the pinched countenance, the emaciation of the entire body, and the cough which had set in in the incipient stage of the disease, believed in galloping consumption, and proposed cod-liver oil. M. Herard, who had long been acquainted with the patient, found it impossible to adhere to any of these views, and, moreover, unable to venture, under the existing circumstances, on the exhibition of cod-liver oil, surmised that the previous protracted abstinence might possibly have some share in the aggravation of the symptoms, and determined upon trying the effects of nutriment. He found it almost impossible at first to carry out this plan, and it was with the utmost difficulty that a few drops of iced beef-tea were swallowed. He succeeded by dint of perseverance, however; and when the food remained on the stomach, and in proportion to its increase, the pulse fell from 145 to 130, 120, 115 and 100; the delirium yielded, and, in short, the patient recovered.

M. Herard is convinced that similar cases are not of unfrequent occurrence, and that the dangerous symptoms of the ataxic form of typhoid are often induced by the strict abstinence previously enforced. In a highly interesting paper on the subject, M. Marotte has established that vomiting, diarrhœa, and delirium, more especially the latter, are characteristic of starvation. In a lecture recently published, M. Trousseau already pointed out the striking analogy existing between the more serious symptoms of typhoid and those of autophagy consequent on protracted abstinence. The valuable experiments of M. Chossat may further be adduced in illustration of the theory which accounts for this extremely important fact, and must lead to a complete change in the treatment of typhoid fever.

"The expression we advisedly use is treatment, not diet. Nutriment here must be viewed not as an adjuvant, but as the principal medical agent. It has been objected that if food be exhibited, indigestion and emesis must follow. This is correct after protracted abstinence, and proves the necessity of early alimentation, otherwise the digestive powers of the stomach become impaired and the food is rejected. Opponents of the method further urge the impossibility of venturing on the exhibition of nutriment, on account of the deposits which necessarily exist on the mucous surface of the stomach, and poison the breath of the patient by their decomposition. Now these deposits are frequently but one of the consequences of abstinence, and if the tongue and gums are cleansed with a brush impregnated with honey of roses or syrup of mulberries, the sores do not form again after the ingestion of food. M. Herard had recently under his care, at Lariboisiere, patients who have fasted for three weeks, and who displayed marked distaste for any kind of nutriment. The gums were covered with sores, the breath was foul; but after cleansing the mouth and scraping the tongue, food, which these patients were compelled to take, produced its usual salutary effects, and in a few days was accepted with pleasure and with the most beneficial results. M. Marotte relates the case of a young man, aged twenty, who, at first compelled to eat, soon took his food with pleasure, and ultimately recovered in an unhoped-for manner. The propriety of feeding patients suffering from typhoid has also been questioned in another respect; the presence of intestinal ulceration, of tympanitis and diarrhœa had been viewed as a direct counter-indication to the exhibition of nutriment, and as the probable cause of the most perilous symptoms in case this method was resorted to. This fear is entirely chimerical. You must not, moreover, forget that the cachectic condition of the patients is the greatest possible obstacle to the healing of the ulcers, and that the latter are portals through which poisonous principles will most readily be admitted. Subjects affected with intestinal ulcerations should be fed, and the ulcers, nevertheless, decrease in size, and heal in the same manner as bed-sores, so common under similar circumstances, yield to the influence of generous diet. Some short time ago, a woman was admitted into the Hotel-Dieu, on the twentieth day of a typhoid fever complicated by extensive mortification in the region of the sacrum. Nutriment, appropriate in nature and in quantity, was gradually exhibited, and the wound speedily lost its pale aspect, assumed a more healthy hue, granulated, and healed. Had abstinence from food been here persevered in, she would very probably have perished; but a contrary course was followed, and she recovered rapidly. Another beneficial effect of nutriment is to shorten the duration of the convalescence, which formerly was interminable after putrid fever. Patients, who have received adequate support during the progress of typhoid, have been known to pass without any transition from disease to health, and walk in the garden of the hospital on the very first day they left their bed.

"It is not unimportant to inquire what should be the nature of the nutriment allowed. It was formerly the custom to exhibit food when only the feverishness had subsided, and it sometimes unfortunately happened that the delay was so long as to render the food superfluous. Other practitioners prescribe broth, under the impression that broth is sufficient support to the system. Broth is doubtless a nutrimental substance; we are all acquainted with its restorative powers, but we must not exaggerate its value. M. Bouchardat demonstrates that a quart of broth contains but six drachms of solid nutriment, two of which are saline ingredients; substract from the remaining four drachms a certain amount which passes through the kidneys, and you will doubtless agree with me that the residue affords but insufficient support to the system.

"M. Herard proceeds then to describe his mode of administering food in typhoid. Soups are, in his opinion, the best articles of diet; egg-flip is often useful, and contains a larger proportion of nutriment especially applicable in the thoracic variety of the disease. Jellies are also advantageous, and when, on account of their volume, soups are not easily digested, the professor, even at an early period of the fever, does not hesitate to recommend the suction of mutton-chop. Patients whose stomach rejects the weakest broth, frequently digest with facility a small piece of broiled beef or mutton. He is no friend of the debilitating *tisanes* and diet-drinks usually prescribed, but agrees with M. Monneret in the utility of wine, as a stimulant of the vital powers. The beverage he recommends is weak wine and water, and, in addition, eight ounces of Bordeau or bark wine, to be taken in enemas if necessary. When the digestive powers of the stomach have been much impaired, he conceives that pepsine, acting as a kind of ferment, promotes the assimilation of the food and gives the gastric viscera time to recover their secretive action, the patient in the meanwhile, not suffering from the effect of injurious abstinence. Fifteen grains of pepsine may therefore be exhibited in a wafer with animal food.

"In addition to these physical restoratives, M. Herard has recourses to moral agency. The greater number of individuals suffering from typhoid fever in the hospitals are young people of both sexes, not only strangers in Paris, but often foreigners. Their isolated condition, combined with the knowledge that they are laboring under serious illness, has much to do with the low condition into which they speedily fall. Hence the importance of encouraging such patients by a kindness of manner and of language calculated to improve their moral condition, and to counteract the unfavorable influence exercised upon their system by the distressing circumstances under which they happen unfortunately to be placed."—*Journ. Pract. Med. and Surg.*, May, 1861.—*Am. Med. Jour.*

